# Prediction of Forelimb Reach Results From Motor Cortex Activities Based on Calcium Imaging and Deep Learning

**DOI:** 10.3389/fncel.2019.00088

**Published:** 2019-03-12

**Authors:** Chunyue Li, Danny C. W. Chan, Xiaofeng Yang, Ya Ke, Wing-Ho Yung

**Affiliations:** School of Biomedical Sciences and Gerald Choa Neuroscience Centre, Faculty of Medicine, The Chinese University of Hong Kong, Shatin, Hong Kong

**Keywords:** motor cortex, two-photon imaging, movement prediction, deep learning, convolutional neural network

## Abstract

Brain-wide activities revealed by neuroimaging and recording techniques have been used to predict motor and cognitive functions in both human and animal models. However, although studies have shown the existence of micrometer-scale spatial organization of neurons in the motor cortex relevant to motor control, two-photon microscopy (TPM) calcium imaging at cellular resolution has not been fully exploited for the same purpose. Here, we ask if calcium imaging data recorded by TPM in rodent brain can provide enough information to predict features of upcoming movement. We collected calcium imaging signal from rostral forelimb area in layer 2/3 of the motor cortex while mice performed a two-dimensional lever reaching task. Images of average calcium activity collected during motion preparation period and inter-trial interval (ITI) were used to predict the forelimb reach results. The evaluation was based on a deep learning model that had been applied for object recognition. We found that the prediction accuracy for both maximum reaching location and trial outcome based on motion preparation period but not ITI were higher than the probabilities governed by chance. Our study demonstrated that imaging data encompassing information on the spatial organization of functional neuronal clusters in the motor cortex is useful in predicting motor acts even in the absence of detailed dynamics of neural activities.

## Introduction

A central question in neuroscience is how the motor cortex encodes movements (Georgopolous et al., 1988; Russo et al., 2018). One commonly used method to address this question is to implant one or several microelectrode arrays in the motor cortex and record electrophysiological signals while the subject repeats the same behavior task, such as center-out reach task and food reaching task (e.g., Sussillo et al., 2015; Li et al., 2017). Although in the past decades this approach has generated significant amount of information in understanding the relationship between motor cortex and behavior, the results obtained mainly describe the temporal evolution of neural activity during movement but provide limited spatial organization information of the neurons involved (O’Shea et al., 2017). Also, extracellular electrode recording is biased toward highly active neurons. On the other hand, two-photon microscopy (TPM) can record faithfully at single-cell spatial resolution from a much larger population of neurons, regardless of their level of activities, and for an extended period of time spanning across weeks. These advantages make TPM a powerful tool to study fine spatial organization of neuronal ensemble in motor cortex in relation to behavior. Indeed, recent imaging studies have shown that micrometer-scale spatial organization may be a characteristic and plays crucial role in movement encoding. For example, ∼70 μm region-specific functional clusters in layer 2/3 of the motor cortex were revealed while mice conducted one-dimensional lever reaching task. Moreover, ensemble and individual activities of task-related cells within the cluster can more accurately reconstruct lever movement trajectories than those of task-related cells outside the cluster (Hira et al., 2013). A more recent study used retrograde pseudotyped lentivirus to label corticospinal neurons (CSNs) that send direct projection to the spinal cord, and found that subgroups of CSNs are activated in specific cortical locations and in precise temporal orders during a food reaching task (Wang et al., 2017).

In primates, premotor areas are involved in movement planning, whereas the primary motor cortex is more likely to be active during movement execution. It has been shown that unilateral lesion or inactivation of premotor areas during movement planning interferes with upcoming forelimb movements in the contralateral direction without impairing movements (Cisek and Kalaska, 2005; Churchland and Shenoy, 2007). Therefore, it may be feasible to predict features of upcoming movement by means of neural activity recorded in motor planning period (e.g., Filippini et al., 2017, 2018). In fact, such knowledge has been put into application in the field of brain-computer interface (Andersen et al., 2014). Several types of neuroimaging data, including functional magnetic resonance imaging (fMRI), functional near-infrared spectroscopy (fNIRS), and wide field calcium imaging, have been used to predict the motion intent or to infer the cognitive state. These imaging signals mainly reflect the input and intracortical processing of a given brain area, usually in the scale of millimeters or hundreds of micrometers (Logothetis et al., 2001). However, whether the spatial organization information in micrometer-scale during motor planning could be used to predict the upcoming motion states or motion dynamics has not been studied.

In rodents, the rostral forelimb area (RFA) has been considered to be a premotor area related to the planning and execution of forelimb movement (Rouiller et al., 1993). In this study, we trained mice to do a novel two-dimensional (2D) lever reaching task and simultaneously recorded calcium imaging signals from layer 2/3 of the contralateral RFA. We used the mean calcium image of movement planning period to predict the maximum reaching location and the reaching outcome, that is, success or failure. We employed a deep convolutional neural network (CNN) named Resnet, which has been shown to achieve outstanding performance in object recognition (He et al., 2016). Unlike the commonly used calcium signal analysis method, we did not extract calcium fluorescent activity (ΔF/F_0_). Rather, we let the CNN model to learn the spatial feature of the functional neuronal clusters directly from the data via a hierarchical layer-based structure. To the best of our knowledge, this is the first study to use deep learning method to analyze micrometer-scale calcium imaging data in motor task.

## Materials and Methods

### Animals and Surgery

All procedures were in accordance with protocols approved by the Hong Kong Department of Health and the Chinese University of Hong Kong Animal Experimentation and Ethics Committee. Wild-type C57BL/6 mice were group housed in standard large cages under normal light cycle (12-h light/dark cycle and lights on at 7:00 am). The cages were each enriched with a plastic house, tunnel system, and low-profile running wheel. Behavioral experiments were performed in the light period.

Male adult mice (8–9 weeks old, *n* = 4) were anesthetized with isoflurane and intraperitoneally injected with ketamine (150 mg/kg) and xylazine (10 mg/kg). A subcutaneous injection of carprofen (5 mg/kg) is administered to reduce inflammation. A custom head-plate was glued to the skull and craniotomy (5 mm diameter) was performed over the left RFA. Adeno-associated viruses (AAV) carrying genes for the calcium indicator GCaMP6f (AAV1.Syn.GCaMP6f.WPRE.SV40, Penn Vector Core) were injected in the left RFA of the motor cortex around the coordinate of 1.0 mm anterior and 2.5 mm lateral from bregma (Hira et al., 2013). Two weeks after the AAV injection, a chronic glass imaging window was implanted. The procedures were separated to reduce the progression of bone regrowth under the window at the later stages of training. Gel superglue was applied at the gap between the glass plug and the skull. Buprenorphine and Baytril were injected subcutaneously at the end of surgery.

### Behavior Training Paradigm

**FIGURE 1:**
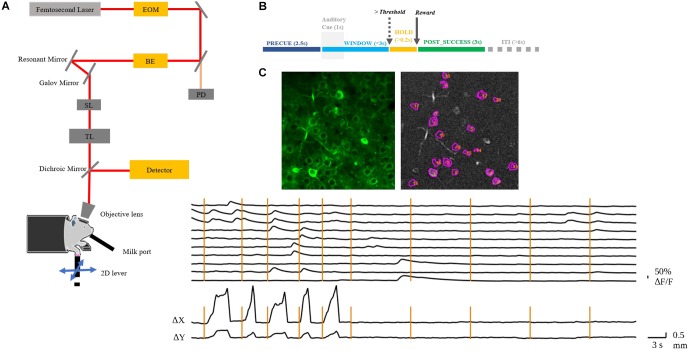
(**A**) Layout of the custom-built TPM. EOM, electro-optic modulator; BE, beam expander; SL, scan lens; TL, tube lens. The mouse with craniotomy was head-fixed under the objective. (**B**) Behavior experiment schema. The mouse was required to hold the lever for 2.5 s to initiate an auditory cue. After the presentation of the auditory cue, the mouse could move the lever in any direction and would receive a chocolate milk reward when the reaching movement exceeded a preset threshold distance from the initial position. The WINDOW for mouse to complete the task was 3 s. If successful, the state was preemptively changed to POST_SUCCESS for reward. (**C**) A representative image of the left RFA with identified neurons in motor cortex layer 2/3 of mouse 9L (top); the normalized Δ*F*/*F* of the numbered neurons and the corresponding lever trajectory indicated as Δ*x* and Δ*y* (bottom). The vertical lines indicate the auditory cue of each trial.

Three days after the window surgery, mice were food restricted and lasted throughout the whole experiment period, maintaining at 85% of their *ad libitum* body weight. Four mice were trained to perform a 2D lever reaching task daily over 17 days (Figure 1A). The lever trajectory was detected in real-time by an independent microcontroller at a rate of 90 Hz. Each trial began at a motor-enforced center position. After contact of the mouse limb with the lever was detected, the animal was required to hold the lever for 2.5 s to initiate an auditory cue. This PRECUE period allowed the mouse to prepare for its movements and the cue signaled the animal to start the task. After cue presentation, the animal was free to move the lever in any direction and was given a chocolate milk reward when the movement exceeded a predetermined threshold distance from the initial position, which increased with training days and was fixed at 3 mm when the expert level was reached. The mouse must complete the task within a fixed WINDOW period of 3 s. If successful, the state was preemptively changed to POST_SUCCESS for reward. On the other hand, the POST_FAILURE state was reached if the window period had elapsed, or if the distance threshold was met but the physical contact with the lever was lost. Generally, the inter-trial interval (ITI) was set as 6 s, and the trial initialization was conducted during this period. However, the ITI would become longer if the mouse refused to touch the lever. The training paradigm is summarized in Figure 1B.

### Two-Photon Calcium Imaging

*In vivo* imaging was conducted by a custom-built, resonant scanner-based TPM (Figure 1A). During the behavioral tests, the mouse was head-fixed under the microscope. Calcium fluorescent signals (512 × 512 pixels per frame) at layer 2/3 of the target RFA (Figure 1C) were acquired at 15 frames per second and were synchronized with the behavioral system by a data acquisition system. In the present study, motion artifacts in calcium imaging data were mainly caused by respiration and cardiac activity. Studies have shown that motion artifacts induced by these physiological processes are restricted mainly in the X-Y plane after using a glass coverslip and head-fixed device (Mohammed et al., 2016). We chose a commonly used method, TurboReg (Thevenaz et al., 1998), to reduce motion artifacts. For each trial, we took the averaged image as the template and registered each frame to the template image. After motion correction, we obtained the mean calcium image of the PRECUE period for each trial. The averaged images were used as the input of the prediction model. Pre-processing of imaging data included resizing and normalization to produce suitable input size and range of intensity required by the deep learning model. The calcium fluorescence changes (Δ*F/F*) were detected by an open source toolbox available online based on a published paper (Pnevmatikakis et al., 2016).

### Data Analysis and Prediction Model

The maximum reaching location (*x_end_*, *y_end_*) was defined as the end point of reaching trajectory during WINDOW period. In our study, each trial began at a motor-enforced center position. We collected $\left(x_{\mathrm{end}}^{n},y_{\mathrm{end}}^{n}\right)$, *n* ∈ [1, *N*] from the *n^th^* trial that received reward. N was the total trial number. For mouse 7N, we categorized the reaching locations into 4 clusters signifying 4 spatial territories. For mouse 9L, we categorized the reaching locations into 3 clusters. Then k-Means method, which is an iterative data-partitioning algorithm that assigns N observations to exactly one of k clusters defined by centroids (Lloyd, 1982), was applied on all data pairs. Here we set k as 4 for mouse 7N and 3 for mouse 9L to make trials that recorded in the same day could at least be separated into two clusters. We labeled each data pair $\left(x_{\mathrm{end}}^{n},y_{\mathrm{end}}^{n}\right)$, *n* ∈ [1, *N*] according to their clustering results [label ∈ {1, 2, 3, 4}for 4 clusters, label ∈ {1, 2, 3} for 3 clusters]. After collecting all trials’ coordinates of the reach location and clustering all these coordinates by k-Means clustering algorithm, we then used the calcium imaging data of the motor planning period to predict each trial’s maximum reach location category.

The calcium imaging data used in this study were recorded during the behavior training period, and the reward threshold distance was adjusted during this period. For study on the prediction of trial outcome, we need to clearly separate the data sets for SUCCESS and FAILURE trials. Therefore, we applied the following exclusion criteria. First, we excluded a trial if the mouse pushed the lever beyond 0.5 mm during PRECUE period. Second, for trials that the animal did receive reward, we excluded the trial if the reaching distance during the WINDOW period was lower than 2 mm for Mice 7N and 1mm for Mice 9L. Third, for failed trials, we excluded the trial if the lever reaching distance during the WINDOW period was larger than 0.5 mm.

**FIGURE 2:**
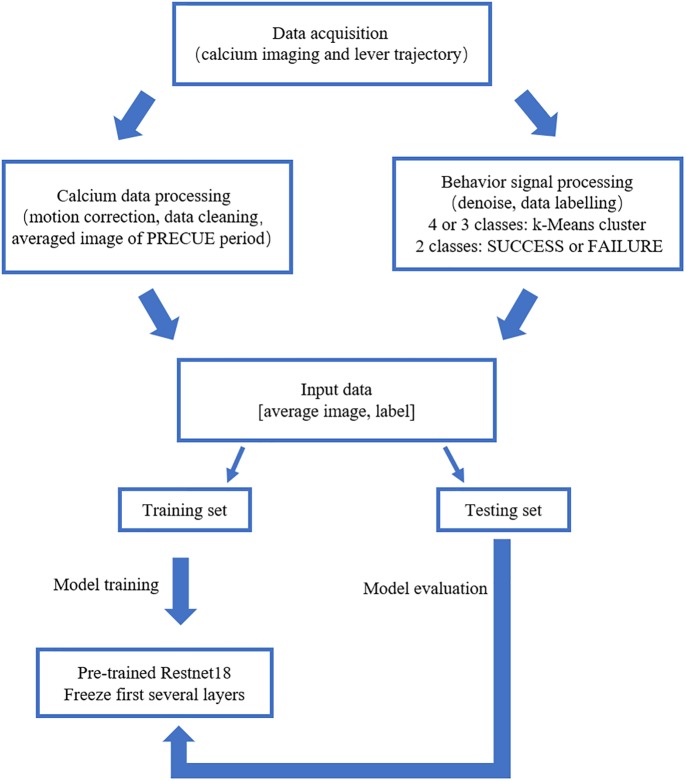
Outline of the data analysis procedure. Calcium imaging data recorded in PRECUE period were collected in synchrony with forelimb movement parameters. TurboReg was used to remove motion artifacts of the imaging data within each trial. Behavioral data were denoised, labeled and k-Means algorithm was used to cluster maximum lever reaching position of all trials. The averaged calcium image were used to predict the result of each trial (SUCCESS or FAILURE) and the maximum lever reaching position. The prediction model was a pre-trained Resnet18. Transfer learning scheme was adopted due to the small sample size. The proportion of the training set and testing was set at a ratio of 4:1.

We built the deep learning prediction model based on a pre-trained Resnet18 (He et al., 2016). The whole set of data was subdivided into training set and testing set in the ratio of 4:1. The training set was used to train the prediction model. The testing set was used to evaluate the performance of the prediction model. Figure 2 summarized the design of the data acquisition and analysis procedures.

### Configuring the Convolutional Neural Network Model

**FIGURE 3:**
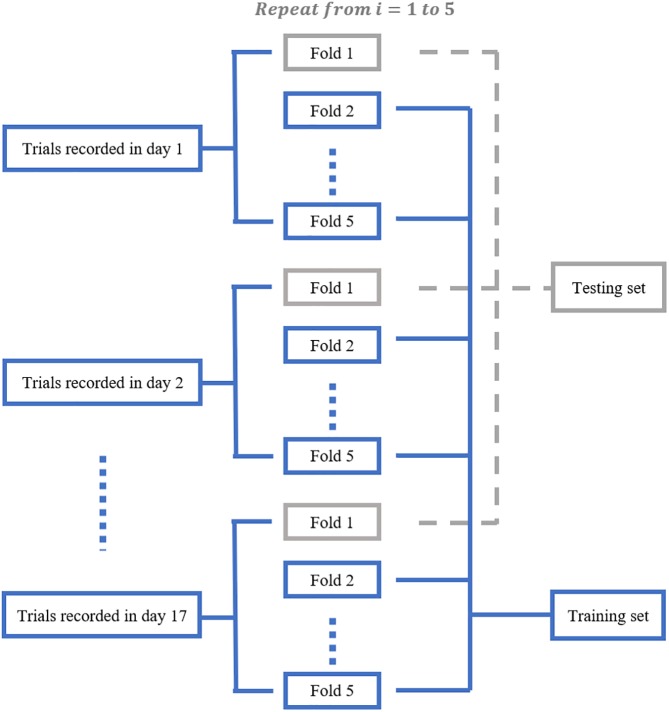
The five-fold data separation procedure. The calcium imaging data recorded in each day was randomly partitioned into 5 portions. One portion was chosen as the testing dataset and the remaining data were used as training dataset. The classification experiments were repeated for 5 times.

We aimed to train a Resnet18 model to map each mean calcium image during the PRECUE period into the corresponding reaching result. When configuring this CNN model, we took into account some specific properties of our input data. First, the calcium imaging signals were collected across days, meaning that there might exist unique 3-dimensional (3D) displacement in the recording area of each recording day. Thus, to make sure that training dataset and test dataset have similar distributions, for each subject, we randomly partitioned the calcium imaging data recorded in each day, rather than all collected data, into 5 portions and took one portion as testing dataset and left all the others as training dataset. Experiments would be repeated for a total of 5 times and the results averaged. This specific five-fold data separation procedure is illustrated in Figure 3. Second, in view of the relatively small sample size available, the strategy of transfer learning was employed in our study. Transfer learning makes use of the knowledge gained while solving one task and applies it to a different task. In this study, Resnet18 was pre-trained on dataset ImageNet, which contains 1.2 million images with 1000 categories. Deep CNN discovers hierarchical feature representations such that higher-level features can be derived from lower-level features. Usually, lower CNN layers are used to extract abstract features like edges, and deeper CNN layers are used to find features that are informative for the target task (Shen et al., 2017). Thus, we froze the initial values of the first several CNN (first 6 layers for mouse 7N and first 13 layers for mouse 9L) and trained the remaining CNN layers with the training set. Also, the number of fully connected layer was adjustable, depending on the task involved.

**FIGURE 4:**
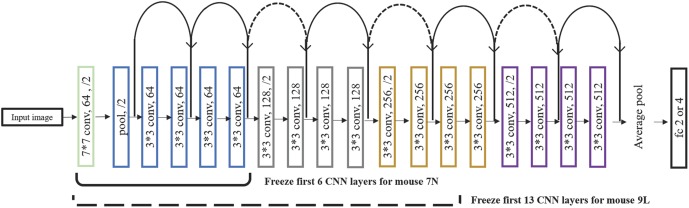
The structure of the Resnet18 employed. Resnet18 was pretrained on the dataset ImageNet, which contains 1.2 million images with 1000 categories. The fully connected layer was changed to 2 to predict the outcome of the trial and to 3 and 4 to predict the maximum lever reaching position. We froze the initial values of the first several CNN (first 6 layers for mouse 7N and first 13 layers for mouse 9L) and trained the remaining CNN layers by using training set. Solid curves represent shortcut connections that performed identity mapping between feature maps that have the same dimensions. Dashed curves represent shortcut connections that performed identity mapping when feature maps have different dimensions.

After a series of testing and optimization, the structure of the specific Resnet18 model shown in Figure 4 was used in the present study. This model started with one convolutional layer with 7 × 7 filters and one pooling layer, mapping 224 × 224 images to 56 × 56 feature maps. This was followed by a sequence of 4 convolutional layers with 3 × 3 filters, and then an average pooling layer and a fully connected layer. Each pair of 3 × 3 filters was added with a shortcut connection. We figured that the imaging data could be used by this CNN model in at least two different ways, namely, to predict the maximum reach location of the successful trials, and to distinguish the successful and failed outcome of the trials.

The optimization method used was Adam (Kingma and Ba, 2014) with a mini-batch size of 16. For mice 7N, the following parameters were used to predict maximum reach location: learning rate was set to 0.00001 and was divided by 10 every 150 epochs; the weight decay was set to 0.0001, and the total epoch number was 300. Moreover, for the outcome of the trials prediction, the weight decay was set to 0.0005, with other parameters remained the same. For mice 9L, the learning rate for maximum reach location was set to 0.00001 and was divided by 10 every 150 epochs, the weight decay was set to 0.00001, and the total epoch number was 300. Similarly, for reach outcome prediction, the weight decay was set to 0.0001, with other parameters are same as above.

Moreover, class activation mapping (CAM) technique (Zhou et al., 2016) was used to visualize the discriminative parts of different categories used by Resnet model for prediction. Let *f_k_* (*x*, *y*) be the task-specific feature map of filter *k* in the last convolutional layer at spatial location (*x*, *y*), and *w_k_* be the corresponding weights of the fully connected layer, which indicates the importance of *f_k_* for different categories. The CAM was calculated as follow:

$$M_{\mathrm{CAM}}\left(x,y\right)=\sum_{k}f_{k}\left(x,y\right)\cdot w_{k}$$

Therefore, *_M_CAM__* could highlight the most informative regions in the image relevant to each category.

In addition, the calcium imaging data collected during the ITI period were used for comparison, as the motor cortical region was presumably in a relatively idle state during this period since the cue for movement task was not yet available. In fact, changes in behavior as a result of learning was obvious in the post-cue period including a reduction in delay in reaching action.

## Results

We trained four mice in total to do the 2D lever reaching task. Three of them successfully learned the 2D reaching task. However, we excluded the data from one mouse that was physically weaker resulting in insufficient number of trials number needed for deep learning analysis. Therefore, two mice (coded 7N and 9L) were included in the present study. The lever trajectory and the corresponding calcium fluorescent traces of individual neurons are shown as an example in Figure 1C.

### Prediction of Maximum Reaching Location

**FIGURE 5:**
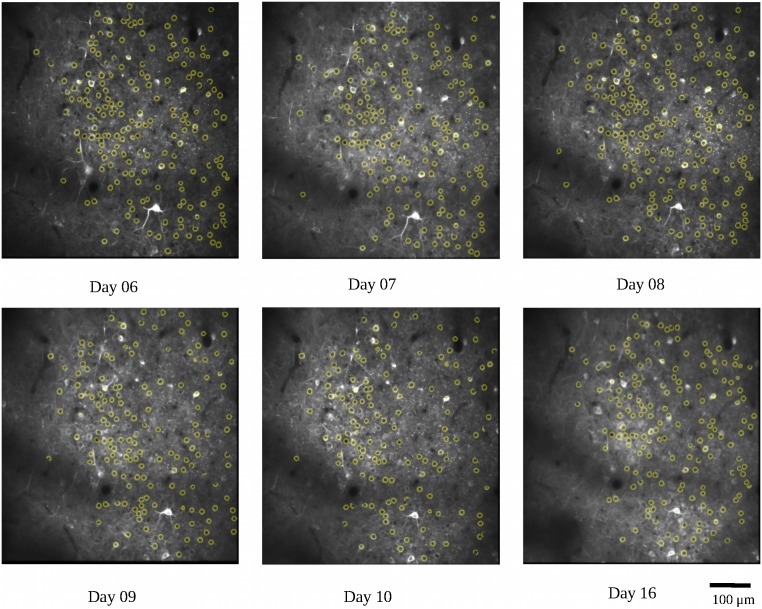
Demonstration of the common neurons (yellow circles) detected in different recording days for mouse 9L. The detected common neurons were compared with neurons revealed in the average image of all recording days.

We first used the calcium imaging data to predict the locations of the lever reach. The imaging data were collected from the two mice (7N and 9L) while they learned the 2D lever reaching task. In this analysis, we only included SUCCESS trials in which the animals received liquid reward, which is considered an indicator of movement intention. At the same time, we could avoid including trials that did not involve planning leading to the failed result. To ensure that the calcium imaging data were from corresponding field and plane, we calculated the common neuron numbers across recording days with respect to neurons detected in the average image of all recording days and removed the recording days with percentages lower than 50%. As an example, the detected common neurons of mouse 9L are shown in Figure 5. There was a total of 320 trials for mouse 7N and 177 trials for mouse 9L

**FIGURE 6:**
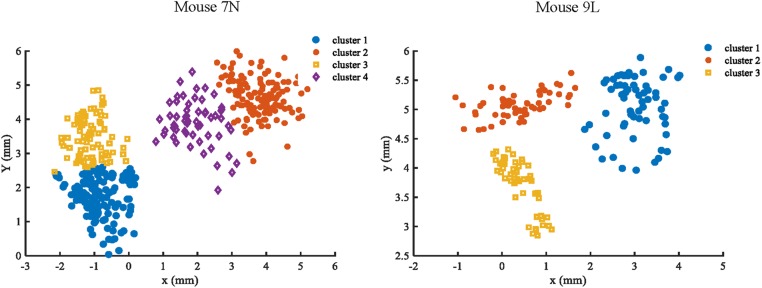
Clustering results of mouse 7N (left) and mouse 9L (right) based on the k-Means method. Different clusters identified are represented by different colors.

To facilitate the assessment of the CNN prediction model, we defined the maximum reach locations by the coordinates of the end point of the lever trajectories (see section “Materials and Methods”), and classified, i.e., labeled, them into different categories. We classified the reach locations of all included trials into 4 clusters for mouse 7N and 3 clusters for mouse 9L to make sure that trials that were recorded in the same day could at least be separated into two clusters. This was to avoid that the Resnet model categorized the results of trials according to the unique 3D displacement characteristics of each recording day. The classification of the maximum reach location was by means of the objective method k-Means clustering. The results of the clustering of the maximum reach locations are shown in Figure 6.

There was a total of 639 ITI for mouse 7N and a total of 516 ITI for mouse 9L. To reduce the impact from the adjacent trial on each ITI due to slow decay of the calcium reporter, we took the averaged image of calcium data collected during a middle 1s of each ITI as the input. For each mouse, the ITI images recorded in each day were chosen randomly into different groups with equal size, and each group is given different cluster labels (1, 2, 3, and 4 for mouse 7N; 1, 2, and 3 for mouse 9L) for maximum reach location prediction. Moreover, the same five-fold cross-validation procedure, transfer learning skill and model parameters were used in this analysis as those used in the RFA group.

The five-fold cross-validation results of the RFA group and ITI group are summarized in Table 1. The training data set for both mice of the RFA group achieved a high level of accuracy on average (>90%) in predicting the maximum reaching location clusters. At the same time, the accuracy for the test data of RFA group are 59.02% for mouse 7N and 64.26% for mouse 9L, while the F-score are 0.56 and 0.62, respectively. Since the probability governed by pure chance in correctly predicting the reach location is 1 in 4, or 25% for mouse 7N and 1 in 3, or 33.3% for mouse 9L, these results indicate that the trained Resnet model possessed reasonably good ability in mapping averaged calcium activity of the RFA to the reaching location in the lever test. In contrast, the accuracy for the test data of ITI group is 23.28% for mouse 7N and 31.90% for mouse 9L, close to the probability of their respective chance level. In addition, we increased the total training epoch number to 600 to address whether the low prediction accuracy for the ITI group was caused by under-fitting of the training data. The obtained five-fold cross-validation results of the ITI group for mice 7N and 9L are summarized in Table 2. As can be seen, the prediction accuracy of mice 7N and 9L are still close to chance level (26.56 and 30.01%, respectively), which do not increase with the rising training accuracy. Taken together, these results indicate that the averaged calcium imaging data of RFA during the preparation period contain specific information that is related to the maximum reach locations.

**Table 1:** Five-fold cross validation results of maximum reach location prediction of the RFA post-cue and ITI group for mice 7N and 9L with epoch number equals to 300.

		RFA post-cue			RFA ITI		
	Fold No.	Accuracy (Training) (%)	Accuracy (Test) (%)	F-score	Accuracy (Training) (%)	Accuracy (Test) (%)	F-score
Mouse 7N	1st	94.05	61.67	0.58	77.76	24.22	0.23
	2nd	92.86	44.44	0.46	72.25	22.66	0.22
	3rd	91.97	60.56	0.57	70.97	22.65	0.22
	4th	90.91	64.18	0.62	70.22	21.87	0.21
	5th	90.77	63.33	0.59	74.96	25.00	0.24
	**Mean**	**92.11**	**59.02**	**0.56**	**73.23**	**23.28**	**0.22**
Mouse 9L	1st	96.43	52.00	0.51	59.92	32.20	0.32
	2nd	94.39	60.00	0.58	62.68	32.03	0.32
	3rd	93.58	67.86	0.63	60.81	35.24	0.34
	4th	93.40	80.64	0.79	62.63	30.47	0.30
	5th	89.47	60.87	0.57	62.95	29.56	0.29
	**Mean**	**93.45**	**64.26**	**0.62**	**61.80**	**31.90**	**0.31**

*Bold values indicate data group names, performance matrices and mean results of five-fold cross validation.*

**Table 2:** Five-fold cross validation results of maximum reach location prediction of ITI group for mice 7N and 9L with epoch number equals to 600.

	Fold No.	Accuracy (Training) (%)	Accuracy (Test) (%)	F-score
Mouse 7N	1st	92.36	26.56	0.25
	2nd	92.64	29.68	0.26
	3rd	93.98	33.59	0.33
	4th	90.69	19.53	0.19
	5th	90.86	23.43	0.22
	**Mean**	**92.11**	**26.56**	**0.25**
Mouse 9L	1st	72.11	27.83	0.27
	2nd	75.77	32.81	0.32
	3rd	76.57	26.22	0.25
	4th	75.67	34.38	0.34
	5th	76.55	28.81	0.29
	**Mean**	**75.33**	**30.01**	**0.29**

*Bold values indicate data group names, performance matrices and mean results of five-fold cross validation.*

### Prediction of Lever Reaching Outcome

**FIGURE 7:**
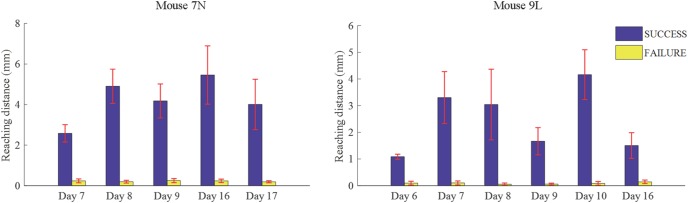
Mean lever reaching distances in each used recording day of mouse 7N (left) and mouse 9L (right).

We considered that a successful trial conducted by the subject was the result of motor planning driven by the motivation to move. In contrast, a failed outcome could reflect lack of sufficient motor planning or motivation. Thus, to provide some insight into these processes, we applied our deep learning model to analyze the activities of the RFA in successful and failed trials. Since the calcium imaging data used in the deep learning model were recorded during the training period, with the reward threshold distance adjusted during the training period, trials with the same reaching distance may result in different outcomes in different training phase. We eliminated such ambiguity by streamlining our data (see section “Materials and Methods”) such that the two data sets were well segregated. The reaching distance of cleaned trials for the two mice are shown in Figure 7. For SUCCESS trials, the mean reaching distance in each recording day was greater than 2mm for mouse 7N and 1mm for mouse 9L. For FAILURE trials, the mean reaching distance in each recording day was lower than 0.5 mm for two mice. Moreover, we randomly deleted trials of each recording day to keep the ratio of successful and failed trials in the range of [1/2, 2], to make sure that the trials of each recording day were balancedly separated into two categories (SUCCESS and FAILURE), and that the Resnet model can learn useful features to distinguish successful and failed trials of each recording day. There was a total of 327 trials (SUCCESS: 186 trials, FAILURE: 141 trials) for mouse 7N, and 247 trials (SUCCESS: 149 trials, FAILURE: 98 trials) for mouse 9L.

The averaged image of calcium signals collected during the middle 1s of each ITI was used as the input for the ITI group. For each mouse, the ITI images recorded in each day were assigned randomly into different groups with equal size, and each group was given different labels (SUCCESS or FAILURE) for reaching outcome prediction. Moreover, the same five-fold cross-validation procedure, transfer learning skill and model parameters were used in the ITI group as in the RFA post-cue group.

Table 3 summarizes the five-fold cross-validation results of both RFA post-cue and ITI group. A high accuracy of prediction was achieved by the model based on the training data set for the RFA group of both mice. Also, the test accuracy is 72.26% for mouse 7N and 69.80% for mouse 9L. The prediction results of RFA group generated by our model are obviously higher than the probability of random guess or pure chance, i.e., 50%. This conclusion is also supported by the results of the sensitivity and specificity of the prediction, as well as F-1 score. However, the accuracy for the test data of the ITI group are still chance level, 49.69 and 48.67% for mice 7N and 9L, respectively. Besides, the higher than pure chance accuracy was not obtained by using a higher epoch number for both two mice (Table 4).

**Table 3:** Five-fold cross validation results on the prediction of reaching outcome of RFA post-cue and ITI group for mice 7N and 9L with epoch number equals to 300.

		RFA post-cue					RFA ITI				
	Fold No.	Accuracy (Training) (%)	Accuracy (Test) (%)	Sensitivity	Specificity	F1-score	Accuracy (Training) (%)	Accuracy (Test) (%)	Sensitivity	Specificity	F1-score
Mouse 7N	1st	94.14	81.36	0.80	0.83	0.78	87.57	44.35	0.45	0.44	0.45
	2nd	93.87	71.42	0.75	0.67	0.67	88.39	46.09	0.42	0.50	0.47
	3rd	93.93	70.23	0.75	0.66	0.67	87.60	49.21	0.50	0.48	0.48
	4th	92.59	68.91	0.74	0.63	0.66	81.57	50.00	0.45	0.55	0.53
	5th	96.76	68.57	0.70	0.67	0.62	86.21	58.80	0.60	0.58	0.57
	**Mean**	**94.23**	**72.26**	**0.75**	**0.69**	**0.68**	**86.26**	**49.69**	**0.48**	**0.51**	**0.50**
Mouse 9L	1st	93.23	71.01	0.76	0.66	0.67	74.46	51.92	0.58	0.46	0.49
	2nd	92.35	67.80	0.79	0.54	0.60	75.00	50.00	0.48	0.52	0.51
	3rd	89.01	73.44	0.83	0.57	0.61	75.92	50.00	0.68	0.33	0.39
	4th	88.46	72.34	0.85	0.57	0.65	77.89	43.22	0.47	0.39	0.41
	5th	83.00	64.61	0.83	0.49	0.60	74.13	48.24	0.42	0.54	0.51
	**Mean**	**89.21**	**69.80**	**0.81**	**0.57**	**0.63**	**75.48**	**48.67**	**0.53**	**0.45**	**0.46**

*Bold values indicate data group names, performance matrices and mean results of five-fold cross validation.*

**Table 4:** Five-fold cross validation results on the prediction of reaching outcome of ITI group for mice 7N and 9L with epoch number equals to 600.

	Fold No.	Accuracy (Training) (%)	Accuracy (Test) (%)	Sensitivity	Specificity	F1-score
Mouse 7N	1st	93.73	46.77	0.42	0.53	0.48
	2nd	96.27	50.00	0.48	0.52	0.51
	3rd	96.06	44.53	0.36	0.53	0.48
	4th	96.85	49.21	0.48	0.50	0.49
	5th	97.86	50.81	0.53	0.48	0.50
	**Mean**	**96.15**	**48.26**	**0.45**	**0.51**	**0.49**
Mouse 9L	1st	85.44	48.08	0.46	0.50	0.49
	2nd	86.54	45.00	0.48	0.42	0.43
	3rd	81.19	46.25	0.55	0.38	0.41
	4th	84.67	52.54	0.53	0.53	0.53
	5th	86.57	49.12	0.53	0.46	0.47
	**Mean**	**84.88**	**48.20**	**0.51**	**0.46**	**0.47**

*Bold values indicate data group names, performance matrices and mean results of five-fold cross validation.*

**FIGURE 8:**
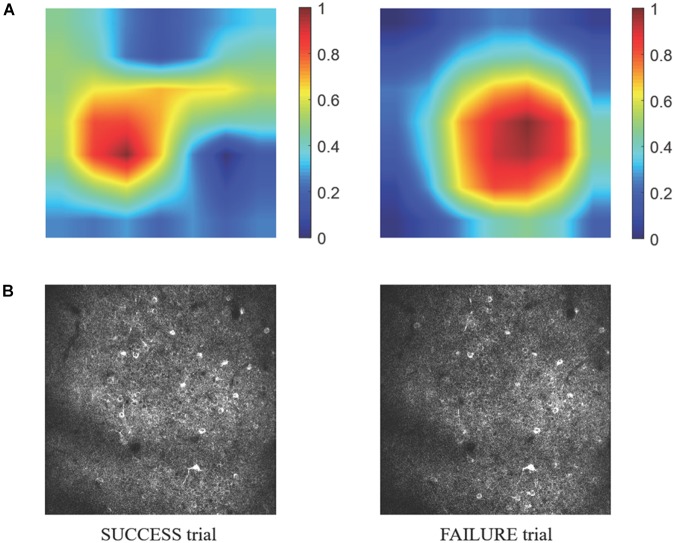
**(A)** The specific discriminative regions used by the prediction model to predict SUCCESS (left) and FAILURE (right) are illustrated by the activation maps. The color indicates the relative value of *M_CAM_*. The higher the *M_CAM_* value, the more important the corresponding area is in prediction. The data are from mouse 9L. The corresponding raw average calcium images are shown in **(B)**.

Because information flow in broad cortical area was reported by using Ca-signal imaging in mouse cerebellar cortex (Kuroki et al., 2018), whether the motion-predictable Ca-signal is recorded only restricted region is crucially important. Thus, we explored what specific discriminative regions were used by the prediction model to predict SUCCESS and FAILURE. We employed a novel technique named CAM (Zhou et al., 2016, see section Materials and Methods), to detect the implicit attention of the prediction model on calcium imaging of the two groups. Figure 8A shows the example of activation maps derived from the prediction model. Figure 8B shows the corresponding raw average calcium images recorded in the same day (the 8th day). It could be observed that the most informative regions in the image relevant to the predicted class are confined to specific sub-regions of the image.

## Discussion

In this study, we recorded calcium signals from layer 2/3 of the RFA of the motor cortex while mice learned a 2D lever reaching task. The image representing the averaged calcium signal collected during the presumed motion planning period was used to predict the maximum reaching location and trial outcome. The prediction results, subject to five-fold cross-validation, are obviously higher than those governed by pure chance. Our study therefore demonstrated that imaging data containing spatial relationship of active neuronal clusters from motor planning region provide non-trivial information related to motion intent and movement target. Significantly, this can be achieved without the need to extract calcium transients, i.e., the usual Δ*F*/*F*_0_ signals that capture neuronal firing dynamics.

Large-scale neuroimaging techniques capable of recording wide areas of human cortex, such as fMRI and fNIRS, have been used to decode behavior, intent, or the cognitive processes (Hong et al., 2015; Zich et al., 2015). Similarly, in rodents, it has been demonstrated that widespread modulation of cortical activation could encode distinct behavior (e.g., Zhu et al., 2018). However, the imaging data recorded in these studies mainly reflect the input or intracortical processing of a given brain area and cannot provide fine scale information on the spatial organization of the microcircuits. Consequently, these techniques are usually used to distinguish very distinct gross motor behaviors or cognitive functions (Poldrack, 2006). At the same time, studies have found that micrometer-scale spatial organization of neurons are associated with both distinct motor activities and fine scale activities (Dombeck et al., 2009; Hira et al., 2013; Wang et al., 2017). Our study supports this notion as our model makes prediction mainly based on spatial features of active neurons at the micrometer scale through deep learning.

The present study also provides some insight into the neurobiology of motor control. In rodents, the functional role of CFA and RFA are still unclear. The classical view is that the CFA and RFA are analogous to the primate primary motor cortex and pre-motor area, respectively (Rouiller et al., 1993) although this view of hierarchical organization has been challenged (Wang et al., 2017). Although the full functional role of RFA is not entirely clear, our results do support the notion that RFA is involved in motor planning, and in line with a recent study suggesting transient and partially distributed neural processing of choice and execution across different subregions of the motor cortex (Morandell and Huber, 2017). It would be interesting to compare the abilities of CFA and RFA activities as derived by TPM in decoding the motion intention and movement-related parameters, based on the deep learning approach, for a better understanding of their functions. One main advantage of TPM is that it can provide single-cell spatial resolution and can faithfully record a larger number of neurons across days. However, the need to combine with genetically calcium indicators limits its application in human, although similar viral delivery methods have already been used in humans for gene therapy (Dunbar et al., 2018; Roelfsema et al., 2018). Nevertheless, TPM has revealed that task-related neuronal groups are activated in specific cortical locations and in precise temporal orders during a learned movement (e.g., Wang et al., 2017). Therefore, studying the spatial activation sequence of movement-related neurons by TPM and its application in movement prediction represents a promising approach in unraveling the neurobiological basis of motor control and motor learning in the future.

Deep learning significantly improves the capability of classical artificial neural networks by incorporating more layers enabling higher levels of abstraction. The particular CNN model ResNet employed in this study is based on a residual learning framework and has been shown to outperform human in object identification (He et al., 2016). A core advantage of deep learning is that instead of using human designed features, it learns task-related features solely from data. In this study, we only resized and normalized all input calcium images to produce suitable input size for the pre-trained CNN model and the same range of intensity for each of the inputs before training the model. We did not provide any guidance except labels.

There are several technical issues that are worth pointing out. First, there were minor 3D displacements in the recording field across days, which may affect the learning by the CNN. To ensure comparisons were made among the same recording fields, we excluded the recording days in which the percentages of common neurons was lower than 50%. As a result, the data from some recording days were removed. Second, a major constraint of deep learning is that it requires a large amount of data to train the model. Consequently, the training process is also time-consuming and it is well-known that full training of a deep CNN is difficult. Thus, we used the scheme of transfer learning in our study. This learning scheme is particularly useful when sufficient data are not available. The pre-trained ResNet18 model with the comprehensively annotated ImageNet data was used for the present task, sparing the need to obtain a much larger volume of calcium imaging data. Third, although our calcium imaging data are different from ImageNet, earlier layers of the pretrained model are expected to extract features, e.g., circles or edges, that also exist in the calcium imaging data. However, the higher-level features of the pretrained model are more specific to the details contained in ImageNet. Therefore, it is necessary to fine-tune the latter CNN layers and derive features that are suitable for calcium imaging data. This approach has been used in medical imaging applications, such as nodule detection and chest pathology identification (Greenspan et al., 2016). Also, based on the results of transfer learning in medical image analysis, fine-tuned CNNs always show better performance than that of the CNNs trained from scratch when the size of training set is small (Tajbakhsh et al., 2016).

A major limitation of the present study is the lack of data in an indifferent brain region during the exact reaching trials for comparison. This is due to the constraint in our 2-photon imaging system preventing us to sample a bigger field of view, or from two fields of view simultaneously. Nevertheless, the spatial calcium activity of RFA during ITI period that was presumably irrelevant or less relevant to movement plan possessed no predictive power. Also, within the RFA, there was spatial sub-regions that were more relevant to prediction outcome. These observations are in line with the conclusion we made.

Despite the limitations of our approach and the modest performance of the prediction model, to the best of our knowledge, this proof-of-principle study is the first work demonstrating that it is possible to infer movement plan from the motor cortex by applying deep learning method on microscopic imaging data. This approach may find applications in different fields including brain machine interface (O’Shea et al., 2017; Pandarinath et al., 2017). Deep learning thus represents a promising direction for future studies. With respect to further development, increasing the sample size should improve the performance of prediction, and reduction of over-fitting of data, as reflected by the discrepancy between the predicting power of the training and testing data sets. Furthermore, since firing dynamics of neuronal ensemble play a significant role in encoding movements (Masamizu et al., 2014; Peters et al., 2014; Li et al., 2017), incorporating information in the time domain would be useful for generating more detailed information, such as the movement trajectory, which was not addressed in the present study. Since the CNN model can automatically learn high-level abstractions from the data, studying the feature map of the CNN layers may also provide insight into movement planning and coding processes.

## Author Contributions

CL, DC, YK, and W-HY designed the study. CL, YK, and W-HY analyzed the data and wrote the manuscript. DC and XY conducted the animal experiments.

## Conflict of Interest Statement

The authors declare that the research was conducted in the absence of any commercial or financial relationships that could be construed as a potential conflict of interest.
